# Potential role of eNOS and EDN-1 gene polymorphisms in the development and progression of retinopathy of prematurity

**DOI:** 10.1186/s12886-023-02810-x

**Published:** 2023-02-24

**Authors:** Aneta Choręziak-Michalak, Anna Gotz-Więckowska, Anna Chmielarz-Czarnocińska, Agnieszka Seremak-Mrozikiewicz, Dawid Szpecht

**Affiliations:** 1 Chair and Department of Ophthalmology, Poznan University of Medical Sciences, ul. Augustyna Szamarzewskiego 84, 60-569 Poznań, Poland; 2 Department of Perinatology and Women’s Diseases, Poznan University of Medical Sciences, Poznan, Poland; 3 Chair and Department of Neonatology, Poznan University of Medical Sciences, Poznan, Poland

**Keywords:** Prematurity, Retinopathy of prematurity, Nitric oxide synthetase, Endothelin, Genetic polymorphisms

## Abstract

The aim of this study was to investigate the association between selected polymorphisms of nitric oxide synthetase (eNOS) and endothelin-1 (EDN-1) with the occurrence and progression of retinopathy of prematurity (ROP). A prospective study was conducted on 90 preterm infants (44 female), comparing 39 cases with ROP and 51 controls without ROP. Patients who developed ROP were further divided into two subgroups—those with spontaneous regression of the disease and those with ROP requiring treatment. We found that preterm infants with TT genotype eNOS 894G > T had a 12.8-fold higher risk of developing ROP requiring treatment (*p* = 0.02). Our results showed that allele T of eNOS894G > T polymorphism was significantly more prevalent in ROP patients requiring treatment (*p* = 0.029). We also investigated preterm infants with TC genotype eNOS − 786 T > C and found an 8.8-fold higher risk developing of ROP requiring treatment (*p* = 0.021). Our results didn’t show any association between EDN-1 5665G > T polymorphism and ROP development. The eNOS polymorphisms appears to influence incidence of ROP requiring treatment in preterm infants. Future research on single nucleotide polymorphisms may provide important information about the pathogenetic mechanisms underlying the development of ROP.

## Introduction

One of the diseases affecting preterm infants is retinopathy of prematurity (ROP), a disorder of immature retinal vasculature, which can lead to retinal detachment and blindness. Vision loss due to the ROP occurs in approximately more than 20,000 infants in the world per year [1]. Presently, there is still no reliable method to predict which patient will develop serious complications of ROP. Excessive oxygen treatment, as well as low gestational age and birth weight, are associated with the development of ROP, but the pathogenesis and etiology of the disease are not completely understood [1], [2]. According to the latest research, genetic factors may be implicated in the development of the disease [3], [4].

The development of ROP is associated with the first (vaso-obliterative) phase of the disease, leading to the suppression of new vessel formation. Then, the second phase of ROP takes place as the developing retina becomes more metabolically active, resulting in massive hypoxia due to its delayed vascularization. Consequently, the vascular endothelial growth factor (VEGF) becomes upregulated and stimulates pathologic neovascularization [5]. Today, there are new pathogenic hypotheses on the development of ROP, including nitric oxide (NO), which is produced in endothelium by nitric oxide synthetase (eNOS) and acts as a vasodilator [6]. Endothelin (EDN-1), which is also synthesized predominantly in endothelial cells, is the most potent of identified vasoconstrictors. NO and EDN-1 are natural regulators in vascular function, determining the blood flow through vessels that may be involved in angiogenesis [7], [8], [9].

A key mechanism of interaction between NO and EDN-1 is cGMP signaling within the endothelium. In the classic endothelin-1/nitric oxide signaling pathway, EDN-1 stimulates eNOS gene expression, leading to enhanced NO production by human endothelial cells [10]. Human eNOS gene is located on chromosome 7q35-36; it contains 26 exons and 25 introns. The − 786T > C polymorphism of NOS3 gene in the promoter region is a missense mutation, which is located in exon 7 of the gene and replaces thymine with cytosine at position 786. The 896G > T polymorphism of NOS3 gene in the coding region, also localized in exon 7, replaces guanine with thymine at position 894, resulting in a change of the amino acid sequence Glu298Asp [11]. Human EDN-1 gene is located on chromosome 6p23-p24 and consists of 6836 nucleotides [12], [13]. The 5665G > T polymorphism includes transversion of guanine with thymine at position 5665, resulting in a change of the amino acid sequence Lys198Asn [12]. Selected polymorphisms within the coding or promoter region of studied genes may alter eNOS or EDN-1 activity and be associated with the susceptibility of the diseases.

In the ROP development, impaired circulation in the premature retina may lead to ischaemia, predisposing to pathologic neovascularization. As it is known, the EDN-1 and eNOS physiologically interact to regulate vascular development and function. In detail, EDN-1 stimulates eNOS to produce NO. However, NO may also inhibit the production of EDN-1 and modulate its vascular effects [7], [10]. An imbalance between these two mediators is a characteristic for endothelial dysfunction and may be relevant for the pathogenesis of retinal vascular abnormalities in premature infants. The differences in the eNOS and EDN-1 genes, which influence NO and EDN-1 activity, may regulate angiogenesis and play the role in the development of ROP.

Therefore, the aim of this study was to investigate the association between the eNOS and EDN-1 polymorphisms with the development and progression of ROP in the Caucasian population of preterm infants.

## Materials and methods

### Design and sample collection

A study was carried out between January 1, 2014 and January 1, 2016, in the Neonatal Intensive Care Unit at the Clinical Hospital of Gynecology and Obstetrics at Poznan University of Medical Sciences in Poznan, Poland.

The study included a cohort of 90 preterm infants qualified for ROP screening in accordance with the consensus of the Polish Neonatologists and the Pediatric Ophthalmology Section (≤ 33 weeks of gestational age, ≤ 1800 g of birth weight or assessed by the neonatologist as at high risk of ROP) [14]. All patients enrolled in the study were of Caucasian origin and born from a singleton pregnancy. Exclusion criteria for the study were as follows: chromosomal abnormalities, birth from multiple pregnancies, birth from pregnancies complicated by the death of one of the fetuses, death before 40 weeks of postmenstrual age (PMA), and the presence of inherited errors of metabolism.

On the basis of ROP screening results, patients included in the study were divided into the control group (without ROP; n = 51) and the case group, who developed ROP (n = 39). The case group comprised patients with spontaneous ROP regression (n = 25) and patients with ROP requiring treatment (n = 14).

In the study group, we evaluated the allele frequency and genotype distribution of single nucleotide polymorphisms (SNP) in genes encoding for eNOS and EDN1, selected on the basis of their potential involvement in the pathogenesis of ROP. The genetic determination of the overmentioned polymorphisms was carried out in peripheral venous blood (0.5 mL), which was taken directly after the delivery and banked. Genomic DNA was extracted from blood leukocytes using the QIAamp DNA Blood Mini Kit (QIAGEN Inc; Germany). Polymerase chain reaction (PCR) and restriction fragment length polymorphism (RFLP) procedures were performed.

The following variables were also recorded from the medical record: sex, gestational age (GA; weeks), birth weight (BW; grams), Apgar score at the 1st and 5th minute, mode of delivery (vaginal birth or cesarean section), birth asphyxia (defined as Apgar score less than 6 at the 10th minute and pH < 7.0 or blood base excess [BE] < -15 mmol/L in cord blood), duration of mechanical ventilation (days), cigarettes in mother, intrauterine infection (defined as a positive sterile culture originally accompanied by clinical symptoms, or pneumonia developed in the first 48 h after the birth), and other than ROP complications of prematurity, including bronchopulmonary dysplasia (BPD), intraventricular hemorrhage (IVH), and necrotizing enterocolitis (NEC).

### ROP diagnosis and management

According to the ROP screening, preterm infants included in the study (≤ 33 weeks of gestational age, ≤ 1800 g of birth weight or assessed by the neonatologist as at high risk of ROP) [14] underwent a series of eye fundus examinations with binocular indirect ophthalmoscopy. The examinations started from the 4th week of chronological age and were performed every 7–10 days, depending on retinal vascularization and the presence of fundus lesions. The diagnosis of ROP was made when ROP vascular features were found at the junction of vascularized and avascular retina. In accordance with the International Classification of Retinopathy of Prematurity (ICROP), they were classified as ROP stages, including demarcation line (stage 1), ridge (stage 2), extraretinal neovascular proliferation (stage 3), then partial or total retinal detachment (stage 4 and 5) [15], [16]. Additionally, other important features were described in ROP cases, including zone and presence of plus disease.

According to the Early Treatment for Retinopathy of Prematurity guidelines, patients diagnosed with ROP were qualified for treatment in the case of any stage of ROP in zone I with plus disease, stage 3 ROP with no plus disease in zone I, or stage 2 or 3 ROP with the plus disease in zone II [17]. These patients were treated by laser photocoagulation of the peripheral avascular retina or intravitreal administration of anti-VEGF antibody (ranibizumab), within 72 h after the diagnosis. In one patient (2 eyes), anti-VEGF therapy was used as the second line of treatment after laser photocoagulation.

The screening was finished in patients with retinal vascularization reaching zone III or regression of ROP on at least two examinations.

### Studied polymorphisms

Details regarding studied polymorphisms and their selected genotyping procedures are presented in Table 1. The criteria for the selection of candidate genes in our study were their potential involvement in the pathogenesis of ROP.

**Table 1:** Description of studied polymorphisms

Gene symbol	Polymorphism	Sequence of primers	Restriction enzyme	Products
eNOS	*894G > T* (*rs1799983*)	F 5` AAggCAggAgACAgTggATgg A 3` R 5` CCC AgT CAA TCC CTT TggTgC TCA 3`	MboI	GG 248 bp GT 248, 158, 90 bp TT 158, 90 bp
eNOS	*-786T > C* (*rs2070744*)	F 5` CCA CCC TgT CAT TCA gTg AC 3` R 5 TCT CTgAgg TCT CgA AAT CA3`	PdiI	TT 296 bp TC 296, 220, 76 bp CC 220, 76 bp
EDN-1	*5665G > T* **(** ***rs5370*** **)**	F 5’TCA TgA TCC CAA gCTgAAAgg CTA3’ R 5`ACC TTT CTT ggAATg TTT TgA AC3’	NheI	GG 203, 25 bp GT 228, 203, 25 bp TT 228 bp

The *eNOS 894G > T (rs1799983)* polymorphism was identified on the basis of PCR amplification with starters: F5′ AAggCAggAgACAgTggATgg A 3′ and R5′ CCC AgT CAA TCC CTT TggTgC TCA 3. The PCR product (248 bp long) was hydrolyzed with MboI restriction enzyme (Thermo Scientific). The following genotypes were found: GG 248 bp, GT 248, 158, and 90 bp, TT 158 and 90 bp.

For detection of the *eNOS 786T > C (rs2070744)* poymorphism, PCR was amplified with starters: F5′ CCA CCC TgT CAT TCA gTg AC 3′ and R5 TCT CTgAgg TCT CgA AAT CA3. The PCR product (296 bp long) waS hydrolyzed with PdiI restriction enzyme (Thermo Scientific). The following genotypes were obtained: TT 296 bp, TC 296, 220, and 76 bp, and CC 220 and 76 bp.

The *EDN1 5665G > T (rs5370)* polymorphism was identified with starters: F5′TCA TgA TCC CAA gCTgAAAgg CTA3′ and R5′ACC TTT CTT ggAATg TTT TgA AC3′. The PCR product (228 bp long) was hydrolyzed with NheI restriction enzyme (Thermo Scientific) and found the following genotypes: GG 203, 25 bp, GT 228, 203, and 25 bp, and TT 228 bp.

To verify the function of each enzyme, positive and negative controls were included during the restriction enzymatic process.

### Statistical analysis

The results were presented as frequencies and percentages of categorical variables or medians (interquartile range) for non-normally distributed continuous variables, as tested using the Shapiro-Wilk test. The threshold of statistical significance was set as *p* value less than 0.05. To determine the association between ROP and categorical variables, the following tests were performed: the Fisher exact probability test, the Chi-squared test, the Fisher Freeman Halton test, and the Chi-squared test with Yates correction. The Mann-Whitney test was used to analyze differences in non-normally distributed continuous variables. The relative risk of ROP was presented by odds ratios (ORs) with corresponding 95% confidence intervals (CI). Statistical analysis was performed using CytelStudio version 11.1.0 (CytelStudio Software Corporation, Cambridge, Massachusetts, United States) and Statistica version 10 (Stat Soft, Inc., Tulsa, Oklahoma, United States).

## Results

The study group consisted of 90 preterm infants and 39 (43.3%) of them were diagnosed with ROP (72 both eyes; 6 only in a single eye). The median gestational age of the study group was 29 ± 2 weeks (range 22–33) and the median birth weight was 1120 ± 366 g (range 510–2340).

Among patients diagnosed with ROP, 25 patients (44 eyes; 27.7% of total) developed spontaneous regression of the disease and 14 of them required treatment (28 eyes; 15.6% of total), including laser photocoagulation (n = 11; 22 eyes), anti-VEGF injection (n = 2; 4 eyes), or both methods (n = 1; 2 eyes).

The incidence of ROP was negatively correlated with gestational age, significantly higher in patients born from 24 to 28 weeks of gestation than in patients born from 29 to 32 weeks of gestation (*p* < 0.0001). Then, the incidence of ROP was also negatively correlated with birth weight (*p* < 0.0001). ROP was more frequently observed in preterm infants with birth weight less than 750 g. The incidence of ROP was the higher the lower Apgar score in the 1st (*p* < 0.0001) and 5th minute of life (*p* = 0.0002). The diagnosis of ROP was more frequent in patients who also developed IVH (*p* = 0.003) or BPD (*p* < 0.0001). Other features, such as gender, mode of delivery, or presence of intrauterine infection, had no obvious differences between the two groups (*p* > 0.05). The duration (in days) of mechanical ventilation was the only variable that differed statistically between the subgroup of patients with ROP that regressed spontaneously and the subgroup of patients with ROP requiring treatment (*p* = 0.005). Table 2 provides characteristics of the study group.

**Table 2:** Characteristics of patients

Characteristic	No ROP (n = 51)	ROP (n = 39)	*p* value	ROP with regression (n = 25)	ROP with treatment (n = 14)	*p* value
**Sex**			0.977^a^			0.831^c^
Female, n (%)	25 (49.0)	19 (48.7)		12 (48.0)	7 (50.0)	
Male, n (%)	26 (51.0)	20 (51.3)		13 (52.0)	7 (50.0)	
**Gestational age** (week)			< 0.0001^a^			0.218^e^
From (24 + 0) to (28 + 6), n (%)	13 (25.5)	31 (79.5)		18 (72.0)	13 (92.9)	
From (29 + 0) to (32 + 0), n (%)	38 (74.5)	8 (20.5)		7 (28.0)	1 (7.1)	
Median (range)	30 (26–33)	26 (22–31)		27 (23–31)	25 (22–30)	
**Birth weight** (gram)			< 0.0001^b^			0.077^a^
<750, n (%)	0 (0.0)	9 (23.0)		3 (12.0)	6 (43.0)	
750–1000, n (%)	9 (17.6)	16 (41.0)		11 (44.0)	5 (36.0)	
>1000, n (%)	42 (82.4)	14 (36.0)		11 (44.0)	3 (21.0)	
Median (range)	1345 (790–2340)	860 (510–1500)		930 (610–1500)	805 (510–1300)	
**Apgar score** (median and range)						
1st minute	6 (1–10)	4 (1–9)	< 0.0001^d^	5 (1–9)	4 (1–6)	0.656^d^
5th minute	8 (5–10)	7 (1–9)	0.0002^d^	7 (1–9)	7 (4–9)	0.226^d^
**Mode of delivery**, n (%)			0.578^a^			0.999^e^
Vaginal	18 (35.0)	16 (41.0)		10 (40.0)	6 (43.0)	
Caesarean section	33 (65.0)	23 (59.0)		15 (60.0)	8 (57.0)	
**Birth asphyxia**, n (%)	3 (6.0)	5 (13.0)	0.44^c^	2 (8.0)	3 (21.0)	0.329^e^
**Mechanical ventilation** (days), median and range	8 (1–53)	54 (3-146)	< 0.0001^d^	44 (3-101)	80 (3-146)	0.005^d^
**Intrauterine infection**, n (%)	24 (47.0)	22 (56.0)	0.379^a^	14 (56.0)	8 (57.0)	0.789^c^
**Cigarettes in mother**, n (%)	3 (5.9)	3 (7.7)	0.932^c^	3 (12.0)	0 (0.0)	0.540^e^
**IVH**, n (%)	11 (22.0)	23 (59.0)	0.0003^a^	11 (44.0)	12 (86.0)	0.028^c^
**BPD**, n (%)	6 (12.0)	20 (51.0)	< 0.0001^a^	11 (44.0)	9 (64.0)	0.378^c^
**NEC**, n (%)	8 (16.0)	9 (23.0)	0.538^c^	4 (16.0)	5 (36.0)	0.238^e^

Results are expressed as total number of patients (percentage) and median (interquartile range). The following tests were used: ^a^chi^2^ test, ^b^Fisher Freeman Halton test ^c^chi^2^ test with Yate’s correction, ^d^Mann-Whitney test, ^e^Fisher exact test. ROP – retinopathy of prematurity, IVH – intraventricular haemorrhage, BPD – bronchopulmonar dysplasia, NEC - necrotising enterocolitis

The analysis showed a significantly higher prevalence of TT genotype (OR 12.8: 1.149–142.6) eNOS 894G > T gene polymorphism in preterm infants with ROP requiring treatment in comparison to preterm infants with ROP that regressed spontaneously (*p* = 0.02). Similarly, allele T of this polymorphism was more prevalent in ROP patients requiring treatment with statistical significance (OR 3.467: 1.115–10.83, *p* = 0.029). The prevalence of genotype TC (OR 8.75: 1.279–95.1) eNOS − 786 C > T polymorphsim was significantly higher in patients with ROP requiring treatment compared to patients with spontaneous ROP regression (*p* = 0.021).

No statistically significant differences were found between the EDN-1 5665G > T polymorphism and the occurrence of ROP. Table 3 reports the genotype and allele frequencies of eNOS and EDN-1 polymorphisms, comparing patients with and without ROP. Table 4 provides the genotype and allele frequencies of eNOS and EDN-1 polymorphisms, comparing patients with spontaneous regression of the disease and patients with ROP requiring treatment.

**Table 3:** Genotype distribution of the studied polymorphisms in patients with and without ROP. ROP – retinopathy of prematurity. EDN1 - endothelin-1; eNOS - endothelial nitric oxide synthase

Gene	Genotype / Allele	No ROP (n = 51)	ROP (n = 39)	*p* value	OR 95% CI
**NOS3** 894G > T (rs1799983)	GG	30 (58.9)	21 (53.8)	-	References
	GT	17 (33.3)	13 (33.3)	1.000	1.092 (0.395–2.986)
	TT	4 (7.8)	5 (12.9)	0.657	1.786 (0.336–10.04)
	Allele G	77	55	-	References
	Allele T	25	23	0.562	1.288 (0.627–2.635)
**NOS3** -786T > C (rs2070744)	TT	19 (37.3)	16 (41.0)	-	References
	TC	28 (54.9)	18 (46.1)	0.713	0.763 (0.286–2.044)
	CC	4 (7.8)	5 (12.9)	0.877	1.484 (0.266–8.767)
	Allele T	66	50	-	References
	Allele C	36	28	1.000	1.027 (0.528–1.986)
**EDN1** 5665G > T (rs5370)	GG	33 (64.7)	26 (66.7)	-	References
	GT	17 (33.3)	11 (28.2)	0.853	0.821 (0.293–2.245)
	TT	1 (2.0)	2 (5.1)	0.853	2.538 (0.124–154.2)
	Allele G	83	63	-	References
	Allele T	19	15	1.000	1.04 (0.453–2.353)

Results are expressed as total number of patients (percentage). The odds ratio (OR) and 95% confidence intervals (95% CI). GG denotes homozygosity for the G-encoded eNOS *894G > T* polymorphism variant; TT homozygosity for the T-encoded eNOS *894G > T* polymorphism variant; GT heterozygosity for eNOS *894G > T* polymorphism. TT denotes homozygosity for the T-encoded eNOS − 786T *> C* polymorphism variant; CC homozygosity for the C-encoded eNOS *− 786T > C* polymorphism variant; TC heterozygosity for eNOS *− 786T > C* polymorphism. GG denotes homozygosity for the G-encoded EDN1 *5665G > T* polymorphism variant; TT homozygosity for the T-encoded EDN1 *5665G > T* polymorphism variant; GT heterozygosity for EDN1 *5665G > T* polymorphism

**Table 4:** Genotype distribution of the studied polymorphisms in ROP patients with spontaneous regression and in ROP patients with treatment. ROP – retinopathy of prematurity. EDN1 - endothelin-1; eNOS - endothelial nitric oxide synthase

Gene	Genotype / Allele	No treatment (n = 25)	Treatment (n = 14)	*p* value	OR 95% CI
**eNOS3** 894G > T (rs1799983)	GG	16 (64.0)	5 (35.7)	-	References
	GT	8 (32.0)	5 (35.7)	0.595	2.000 (0.340–11.54)
	TT	1 (4.0)	4 (28.6)	**0.020**	12.8 (1.149–142.6)
	Allele G	40	15	-	References
	Allele T	10	13	**0.029**	3.467 (1.115–10.83)
**eNOS3** -786T > C (rs2070744)	TT	14 (56.0)	2 (14.3)	-	References
	TC	8 (32.0)	10 (71.4)	**0.021**	8.75 (1.279–95.1)
	CC	3 (12.0)	2 (14.3)	0.456	4.667 (0.226–83.4)
	Allele T	66	50	-	References
	Allele C	36	28	1.000	2.571 (0.878–7.512)
**EDN1** 5665G > T (rs5370)	GG	14 (56.0)	12 (85.6)	-	References
	GT	10 (40.0)	1 (7.2)	0.065	0.117 (0.002–1.093)
	TT	1 (4.0)	1 (7.2)	1.000	1.167 (0.014–98.04)
	Allele G	38	25	-	References
	Allele T	12	3	0.257	0.38 (0.063–1.627)

Results are expressed as absolute number of patients (percentage). The odds ratio (OR) and 95% confidence intervals (95% CI). GG denotes homozygosity for the G-encoded eNOS *894G > T* polymorphism variant; TT homozygosity for the T-encoded eNOS *894G > T* polymorphism variant; GT heterozygosity for eNOS *894G > T* polymorphism. TT denotes homozygosity for the T-encoded eNOS − 786T *> C* polymorphism variant; CC homozygosity for the C-encoded eNOS *− 786T > C* polymorphism variant; TC heterozygosity for eNOS *− 786T > C* polymorphism. GG denotes homozygosity for the G-encoded EDN1 *5665G > T* polymorphism variant; TT homozygosity for the T-encoded EDN1 *5665G > T* polymorphism variant; GT heterozygosity for EDN1 *5665G > T* polymorphism

## Discussion

Retinopathy of prematurity is a potentially blinding disease of the immature retina, characterized by the proliferation of abnormal fibrovascular tissue at the border of the vascularized and non-vascularized retina. The main risk factors, such as gestational age and birth weight, are inversely correlated with the development of ROP, as we confirmed in this study [1], [5]. We also found a statistically significant association between ROP development and Apgar score in the 1st and 5th minute, prolonged mechanical ventilation, the occurrence of intraventricular hemorrhage (IVH) and bronchopulmonary dysplasia (BPD), which was also suggested in other studies [18]. However, the pathogenesis of the disease is not fully understood. Thus, the identification of genetic factors predisposing to ROP could explain the mechanisms underlying the disease and predict its clinical course in patients with individual genotypes.

Nitric oxide (NO) is known as a key signaling molecule in numerous physiological processes, regulating vascular tone control and remodeling [19]. It is endogenously synthesized from L-arginine by nitric oxide synthetase (NOS), a family of enzymes that comprises three isoforms: neuronal NOS (nNOS, NOS1), inducible NOS (iNOS, NOS2), and endothelial NOS (eNOS, NOS3). The endothelial NO is produced in the vascular endothelium and acts as a vasodilator, relaxes smooth muscles and facilitates improved blood flow through vessels. On the other hand, endothelin-1 (EDN-1), which is also synthesized predominantly by endothelial cells, is one of the most effective of identified vasoconstrictors [8].

NO and EDN-1 are important regulators in vascular function, determining the blood flow through vessels that may be involved in angiogenesis. ET-1 stimulates eNOS to produce NO, and then NO triggers the gene expression and activation of several angiogenic and proliferation-inducing factors, including VEGF [7]. In the view of the key role of eNOS and EDN-1 in vasculo- and angiogenesis, we investigated the association between selected polymorphisms of their genes and the development of ROP.

In our study, we reported that preterm infants with TT genotype eNOS 894 G > T had a 12.8-fold higher risk developing of ROP requiring treatment (OR 12.8: 1.149–142.6, *p* = 0.02). Moreover, we found that allele T of eNOS 894 G > T polymorphism was significantly more prevalent in ROP patients requiring treatment (OR 3.467: 1.115–10.83, *p* = 0.029). We also investigated preterm infants with TC genotype eNOS − 786 T > C and found they had an 8.8-fold higher risk developing of ROP requiring treatment (OR 8.75: 1.279–95.1, *p* = 0.021). However, we didn’t find any associations between genotypes or allele frequencies of EDN-1 5665G > T polymorphism and ROP development, including advanced ROP requiring treatment.

According to the literature, contradictory conclusions were reported regarding eNOS gene polymorphisms and ROP. Thus, we aimed to investigate the association between eNOS 894 G > T and eNOS − 786 C polymorphisms with ROP development in our study. Similarly to our results, Yalamandra et al. reported that T allele of eNOS 894 G > T polymorphism was a significant risk factor for the development of ROP in both the Caucasian and African American premature infants [20]. They also found that the frequencies of C allele eNOS − 786T > C were significantly higher among ROP infants in both studied groups. In another study, Yu et al. showed a significant association between − 786 C allele and ROP susceptibility in the study group of 118 cases (with ROP) compared with 134 controls (without ROP) [21]. Their main finding was that − 786 C allele might have a protective effect for ROP. In their study, all genotypes of -786 T > C and 894 G > T had no significant association with ROP development. However, they also showed that the interaction of -786CC and 894GT with oxygen therapy duration (< 17 days) were both protection factors against ROP. Moreover, Ilguy et al. showed that the polymorphism − 786 C > T was associated with severe ROP and the presence of T allele in the studied polymorphism increased ROP severity and treatment requirement in the Turkish population [22]. Similarly, Poggi et al. carried out a retrospective study to evaluate the 894T and − 786 C eNOS polymorphisms [23]. In the first of studied polymorphisms, they found a higher prevalence of GT + TT genotypes in infants with ROP compared to those without ROP, with no significant differences in the genotype distribution in the second of them.

However, the previous study by Rusai et al. compared − 786 C > T polymorphism in NOS gene in ROP cases with and without treatment, indicating no association with ROP severity in the Hungarian population [24]. Finally, Gohari et al. in the systematic evaluated that 894 G > T and − 786 C > T polymorphisms were not associated with the risk of ROP development [25].

The association of EDN-1 5665G > T polymorphism with ROP has not been investigated before. According to the literature, Li et al. found that EDN-1 5665G > T polymorphism was significantly correlated with the increased risk of preeclampsia [26]. The EDN-1 5665G > T polymorphism was studied by Szpecht et al. in preterm infants with intraventricular hemorrhage, but no association was found [27]. In our study, we found no significant differences in genotype distribution or allele frequency of EDN-1 5665G > T polymorphism in preterm infants with and without ROP, when comparing ROP patients with and without treatment.

There are several limitations of our study that should be highlighted. We cannot exclude the possibility that the relatively small group of patients and especially those who developed ROP may prohibit the detection of the existing associations. Thus, the investigation should be performed in a larger sample. In our study, we considered a limited number of variants from a small number of candidate genes, selected on the basis of their potential involvement in ROP pathogenesis. Future studies using innovational technologies such as the next-generation sequencing (NGS) based approach may lead to a revolutionary advance in genetic research, with the possibility to discover a large number of novel genetic variations from targeted regions or even whole genome sequencing. Kim et al. were the first who present a study based on whole exome sequencing in ROP patients, but no single genes reached genome-wide significance associated with a severe phenotype of ROP. However, they suggested potential pathways that may be important in the pathogenesis of ROP, including smooth endoplasmatic reticulum and vitamin C metabolism pathway [28].

To conclude, we investigated preterm infants with TT genotype eNOS 894G > T or TC genotype eNOS − 786T > C and foun they had an increased risk developing of ROP requiring treatment. Moreover, we found that allele T of eNOS 894G > T polymorphism was significantly more prevalent in ROP patients requiring treatment. We didn’t confirm any association between EDN-1 5665G > T polymorphism and ROP. A growing body of evidence suggests that the pathogenesis of ROP is associated with heterogenous retinal disorders, including those in the nitric oxide pathway. Future research on SNPs may provide important information about the pathogenetic mechanisms underlying the development of ROP.

## Data Availability

The datasets generated during and/or analysed during the current study are not publicly available, but are available from the corresponding author on reasonable request.
